# GPS mobility as a digital biomarker of negative symptoms in schizophrenia: a case control study

**DOI:** 10.1038/s41746-019-0182-1

**Published:** 2019-11-08

**Authors:** Colin A. Depp, Jesse Bashem, Raeanne C. Moore, Jason L. Holden, Tanya Mikhael, Joel Swendsen, Philip D. Harvey, Eric L. Granholm

**Affiliations:** 1 Department of Psychiatry, UC San Diego, La Jolla, CA USA; 2 Psychology Service, VA San Diego, San Diego, CA USA; 3 Johns Hopkins School of Nursing, Baltimore, MD USA; 4 National Center for Scientific Research, University of Bordeaux (UMR 5287); EPHE PSL Research University, Bordeaux, France; 5 Department of Psychiatry and Behavioral Sciences, University of Miami, Miami, FL USA

**Keywords:** Health care, Medical research

## Abstract

Mobility is an important correlate of physical, cognitive, and mental health in chronic illness, and can be measured passively with mobile phone global positional satellite (GPS) sensors. To date, GPS data have been reported in a few studies of schizophrenia, yet it is unclear whether these data correlate with concurrent momentary reports of location, vary by people with schizophrenia and healthy comparison subjects, or associate with symptom clusters in schizophrenia. A total of 142 participants with schizophrenia (*n* = 86) or healthy comparison subjects (*n* = 56) completed 7 days of ecological momentary assessment (EMA) reports of location and behavior, and simultaneous GPS locations were tracked every five minutes. We found that GPS-derived indicators of average distance travelled overall and distance from home, as well as percent of GPS samples at home were highly correlated with EMA reports of location at the day- and week-averaged level. GPS-based mobility indicators were lower in schizophrenia with medium to large effect sizes. Less GPS mobility was related to greater negative symptom severity, particularly diminished motivation, whereas greater GPS mobility was weakly associated with more community functioning. Neurocognition, depression, and positive symptoms were not associated with mobility indicators. Therefore, passive GPS sensing could provide a low-burden proxy measure of important outcomes in schizophrenia, including negative symptoms and possibly of functioning. As such, passive GPS sensing could be used for monitoring and timely interventions for negative symptoms in young persons at high risk for schizophrenia.

## Introduction

There continues to be a great need for objective, easily collected measures of symptoms and functional outcomes in schizophrenia research^1^. Prior work with ecological momentary assessment (EMA), delivered through mobile technology, supports the potential of this method for collecting real-time data on symptoms, social participation and other domains^2–4^. However, EMA involves participant burden (e.g., the need for frequent prompted responding on the devices) and, while feasible and acceptable, modal data collection periods in EMA studies are 1–2 weeks in duration^5^. Passive sensors embedded in mobile devices may help gather objective information pertinent to symptoms and functioning, in particular mobility data gathered through global positioning system (GPS) sensors. The purpose of this study was to: (a) validate mobility indicators derived from GPS relative to EMA data based on reports of physical location; (b) compare GPS-derived mobility between people with diagnoses of schizophrenia and healthy comparators, and (c) examine associations within the group with schizophrenia between GPS-derived mobility information and in-lab data collected on symptoms, cognitive performance, and functional measures.

GPS can provide indices of distance traveled from home, time spent at home, locations visited, and variability of all of these indicators. GPS has been employed in public health and ecology research to examine mobility^6^ and community participation and constructs such as “life space”^7^. Life space is the pattern and degree to which an individual moves in their environment, emanating from their home^8^. Persons with greater disability and cognitive impairment tend to travel smaller distances from home as measured by GPS data^9–12^, yet this construct has not been empirically examined in schizophrenia either through questionnaire or GPS data. Nonetheless, related functional constructs in schizophrenia such as community participation and mobility have been evaluated in schizophrenia research through self-reported and informant-based surveys, and reduced mobility indexed by these constructs are predicted by cognitive ability, physical illnesses, and negative symptoms^13,14^.

A handful of studies have evaluated the use of GPS in schizophrenia, often in conjunction with other passive sensors, typically with the intention to predict changes in symptoms and other illness-related factors at the individual level^15–18^. To our knowledge, studies have not evaluated person-aggregated GPS data in a case–control design. Given the increasing use of smartphones in people with schizophrenia^19^, there is great potential for monitoring mobility and ultimately for triggering real-time interventions, especially in younger high-risk or recent-onset populations. Thus, while existing studies with GPS in schizophrenia support the great potential for passive sensing, the focus of these investigations has not to date specifically addressed the validity of GPS-derived indicators in relation to other concurrent data on location, compared averaged values to healthy comparators, or identified which aspects of the illness most correlate with GPS metrics.

The purpose of this case–control study is to evaluate GPS mobility indicators, including the proportion of GPS samples found to be at-home and the distance traveled (overall and from home), in a sample of people with schizophrenia compared to healthy individuals. To do so, participants completed a comprehensive battery of neuropsychological, functional and symptom assessments in the lab, and then completed one week of EMA measures of functional participation and location, as well as GPS sensing. With respect to concurrent validity, we hypothesize that GPS metrics of mobility will be associated with EMA-sampled indicators of time spent in and out of the home. We also hypothesize that participants with schizophrenia will have lower mobility than comparators, and that within the schizophrenia sample that GPS data will correspond to in-lab measures of community functioning. Finally, we expect, based on prior work on mobility in schizophrenia, that GPS mobility indicators will be associated with more severe symptoms, worse neurocognition, and reduced community function.

## Results

### Sample characteristics

Demographics and clinical characteristics are presented in Table 1. The healthy comparator and schizophrenia samples were similar in terms of age, gender, and race/ethnicity distribution. As expected, the sample with schizophrenia was less likely to be employed, less likely to be living independently, and had attained a lower level of education. On symptom and other clinical measures administered only to the sample with schizophrenia, mean values of cognitive performance were in the mild range of impairment and levels of psychopathology were also in the mild-to-moderate severity range.

**Table 1:** Sample characteristics (*n* = 142)

	Healthy comparators (*n* = 56)	People with schizophrenia (*n* = 86)	*T* or *X*^2^	*p* Value
Age (M, SD, range)	51.1 (11.0)	52.0 (9.1)	0.5	0.600
	24–65	27–65		
Sex (% female)	35.7%	30.2%	0.5	0.495
*Race/ethnicity*				
Non-Hispanic White	44.6%	38.4%	7.1	0.310
African-American	25.0%	43.0%		
Latino/Hispanic	17.9%	11.6%		
Other	12.5%	6.8%		
Education (M, SD, range)	14.7 (1.7)	13.0 (1.9)	5.4	<0.001
	12–18	8–20		
% Currently employed	58.2%	18.6%	23.4	<0.001
% Living independently	94.5%	70.9%	11.7	<0.001
Global MCCB T-score (M, SD, range)	–	38.3 (6.2)	–	–
		21–53		
SLOF informant composite (M, SD, range)	–	3.7 (0.8)	–	–
ILSS composite (M, SD, range)	–	0.75 (0.09)	–	–
		0.47–0.92		
CAINS total (M, SD, range)	–	19.4 (8.1)	–	–
		5–40		
SANS total (M, SD, range)	–	9.9 (3.7)	–	–
		3–18		
BPRS-positive symptoms (M, SD, range)	–	9.5 (3.8)	–	–
		3–19		
Calgary depression rating scale (M, SD, range)	–	5.5 (4.3)	–	–
		0–15		

*MCCB* MATRICS consensus cognitive battery, *SLOF* specific level of functioning, *CAINS*: calgary assessment interview for negative symptoms, *ILSS* independent living skills survey, *SANS* scale for the assessment of negative symptoms, *BPRS* brief psychiatry rating scale

### GPS values and EMA data

The average adherence to EMA (number of surveys answered divided by total number possible) was high and did not differ between groups (patient mean = 86.4%, SD = 0.15, healthy comparator mean = 85.5%, SD = 0.15, *T*(140) = 0.3, *p* = 0.748). The total number of accurate GPS samples was higher in the patient group (mean = 1320.4, SD = 451.5) vs. the healthy comparators (mean = 1160.8, SD = 480.5; *T*(140) = 2.1, *p* = 0.047). Further analyses indicated that the number of accurate samples was associated with the ratio of samples at-home (rho = 0.492, *p* < 0.001) and distance traveled from home (rho = −0.205, *p* = 0.014). Therefore, we included the number of GPS samples as a covariate in group comparisons.

### Correlations between GPS and EMA indicators

As seen in Table 2, EMA data on time spent at-home and GPS data were strongly associated across the week for the entire sample. Subgroup analyses (data not shown) indicated similarly strong Rho coefficients in healthy comparators and schizophrenia (all *p* values < 0.003). A day-level mixed models indicated a significant main effect for GPS distance traveled per day in predicting same-day EMA time at-home (estimate = 0.07, s.e. = 0.02, *T*(1,866) = 3.9, *p* < 0.001), and the interaction between diagnostic group and GPS day-level metrics were not significant (estimate = 0.02, s.e. = 0.03, *T*(1,866) = 0.7, *p* < 0.451). Similar patterns were observed for other GPS and EMA indicators at the day level. Therefore, at both the week-aggregated and day levels, EMA and GPS indicators of mobility were strongly associated, and this association did not appear to vary in strength across diagnostic groups.

**Table 2:** Spearman correlations between GPS metrics and EMA metrics in total sample (*n* = 142)

	GPS median daily distance traveled (miles)	GPS median daily distance traveled from home (miles)	GPS median percent of samples at home
*Total sample*			
EMA time spent at home in past hour	−0.575 (*p* < 0.001)	−0.623 (*p* < 0.001)	0.684 (*p* < 0.001)
EMA percent of samples at home	−0.551 (*p* < 0.001)	−0.582 (*p* < 0.001)	0.658 (*p* < 0.001)
*Healthy comparators*			
EMA time spent at home in past hour	−0.375 (*p* = 0.004)	−0.422 (*p* = 0.001)	0.546 (*p* < 0.001)
EMA percent of samples at home	−0.366 (*p* = 0.006)	−0.406 (*p* = 0.002)	0.539 (*p* < 0.001)
*Schizophrenia*			
EMA time spent at home in past hour	−0.569 (*p* < 0.001)	−0.601 (*p* < 0.001)	0.681 (*p* < 0.001)
EMA percent of samples at home	−0.550 (*p* < 0.001)	−0.560 (*p* < 0.001)	0.640 (*p* < 0.001)

Spearman Rho value (*p* value); *EMA* ecological momentary assessment

### Comparison between schizophrenia and healthy comparator samples

As hypothesized, people with schizophrenia had lower average distance traveled, distance traveled from home, and a higher rate of samples at-home than did the healthy comparators (Table 3). The magnitude of these differences were large. EMA mobility indicators were also different across groups, and the magnitude of these differences were large. We repeated all GPS group comparisons with the total number of accurate samples as a covariate, and all group comparisons remained significant (all *p* < 0.001). We also repeated the analyses adjusting for educational attainment, employment, and living situation, and all GPS group comparisons remained significant (*p* < 0.001).

**Table 3:** Group comparison of GPS metrics of distance traveled

	Healthy comparators (*n* = 56) M (SD) range	Schizophrenia (*n* = 86) M (SD) range	*Z* ^a^	*p* Value	Cohen’s *d*
*GPS indicators*					
Median daily distance traveled (miles)	23.8 (17.6) 1–79	12.3 (10.4) 1–52	2.5	<0.001	0.80
Median daily distance from home (miles)	19.8 (16.6) 0–77	8.1 (9.0) 0–38	2.3	<0.001	0.88
Percent of samples at home	51.1% (0.38) 0–100%	74.4% (0.25) 0–99%	1.9	0.001	0.72
*EMA indicators*					
Mean time at home during past hour (min)	32.4 (14.6) 1–54	42.89 (10.8) 8–60	1.9	0.001	0.82
Percent of samples at home	45.7% (0.24) 0–88%	62.2% (0.20) 14–100%	1.8	0.003	0.75

^a^Kolmogorov *Z*-test values; unadjusted means and analyses presented

### Associations with demographics, symptoms, and exploratory investigation of subscales in schizophrenia

Neither age nor education were associated with distance traveled, distance away from home, or proportion of time at home (range of Rho = −0.177 to 0.103; *p* value range = 0.085 to 0.552). Similarly, neither gender (*p* value range: 0.463–0.974) nor living situations were associated (*p* value range = 0.081–0.381). The subgroup of unemployed participants with schizophrenia spent more in the home [77% (0.23) vs. 63% (0.32); t(1) = 2.0, *p* = 0.046)] and traveled lower median distance away from home] (7 miles (8.3) vs. 12.3 (11.0), *t*(1) = 2.1, *p* = 0.037)].

**Fig. 1:**
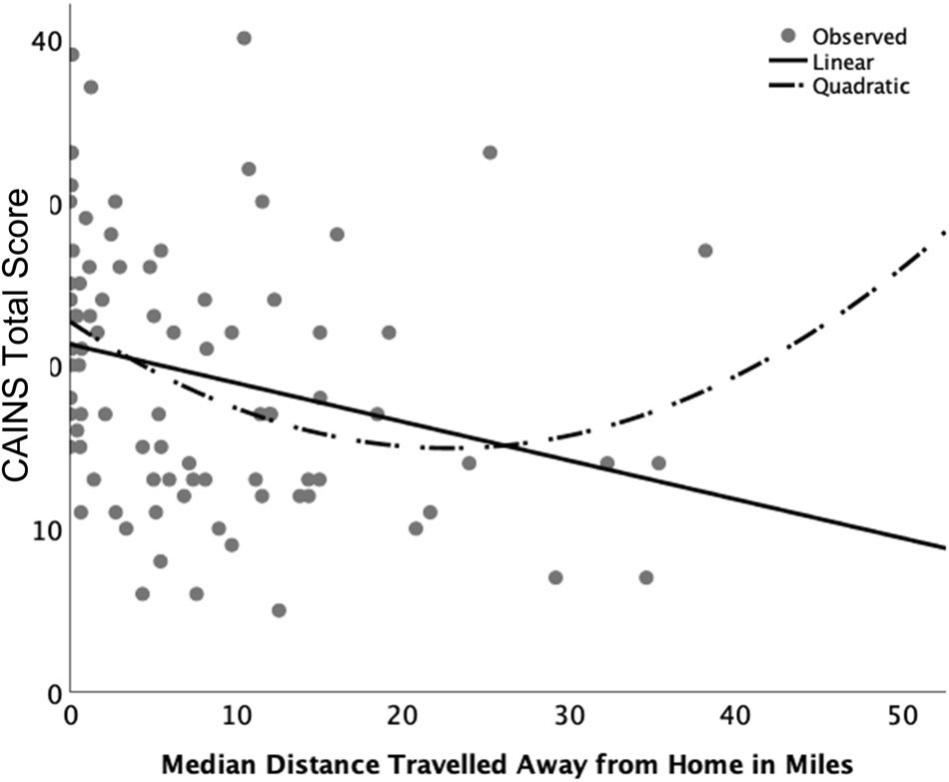
CAINS total score and GPS distance traveled away from home. Scatterplot displays linear and non-linear associations between CAINS total and median distance away from home; tests for coefficients are *Linear:* *R*^2^: 0.07, *F*(1,84) = 6.3, *p* = 0.014; *Quadratic*: *R*^2^: 0.11, *F*(1,83) = 5.0, *p* = 0.009

**Fig. 2:**
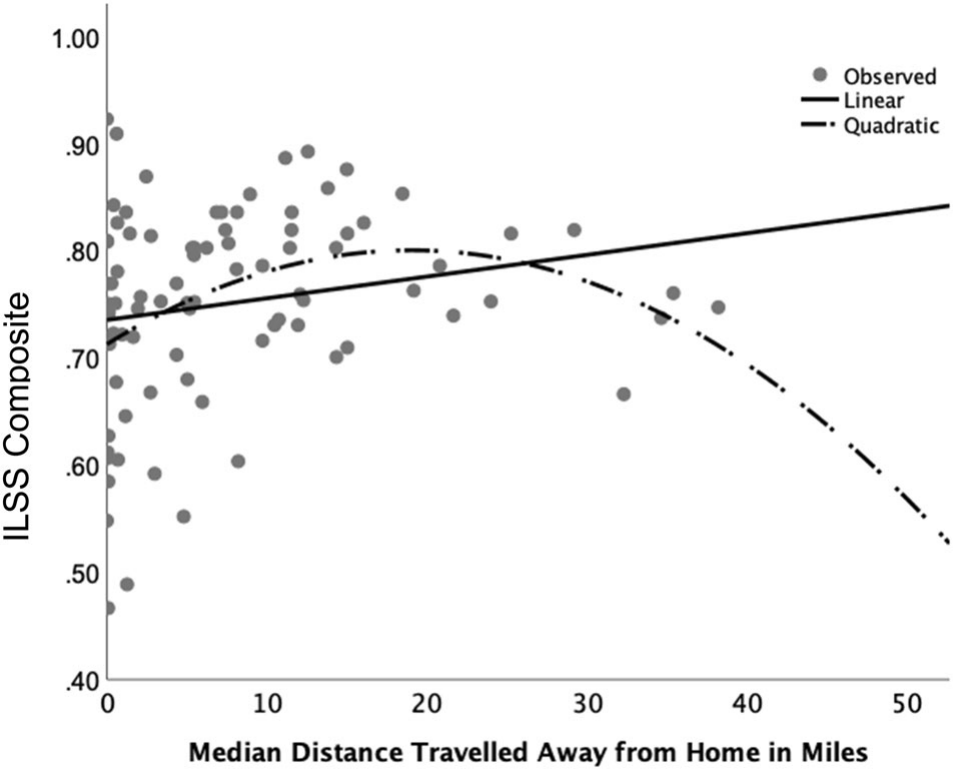
ILSS composite and GPS distance traveled away from home. Scatterplot displays linear and non-linear associations between ILSS composite and median distance away from home; tests for coefficients are *Linear:* *R*^2^: 0.04, *F*(1,84) = 3.5, *p* = 0.065; *Quadratic*: *R*^2^: 0.12, *F*(1,83) = 5.5, *p* = 0.006

As seen in Table 4, modest but significant associations were seen between GPS derived indicators of mobility and negative symptoms and independent living skills survey (ILSS) scores. Neurocognitive ability, depression, positive symptoms, or specific level of functioning (SLOF) scores were not associated with GPS mobility. Applying correction for multiple comparisons, only negative symptoms as measured by the calgary assessment interview for negative symptoms (CAINS) was significant (*p* < 0.007) for either GPS or EMA indicators. Further exploratory analyses examined the subscales of the negative symptom measures. For both CAINS and scale for the assessment of negative symptoms (SANS), subscales pertaining to motivation (CAINS Motivation and Pleasure; rho = 0.341, *p* = 0.001, SANS diminished motivation, rho = 0.343, *p* = 0.001) were the only subscales associated with GPS indicators at the *p* < 0.007 level. Inspection of scatterplots indicated potential nonlinear associations between negative symptoms and functioning. Indeed, quadratic models accounted for the most variation (Figs. 1 and 2), with an observed pattern of steepest association at lowest levels of mobility and weakening association at higher levels.

**Table 4:** Spearman correlations between GPS metrics/EMA metrics and in-lab measures in the sample with schizophrenia

	GPS median daily distance traveled (miles)	GPS median daily distance traveled from home (miles)	GPS percent of samples at home	EMA time spent at home in past hour	EMA percent of samples at home
MCCB global T-score (cognitive ability)	0.078 (0.475)	0.083 (0.446)	−0.039 (0.719)	−0.160 (0.141)	−0.116 (0.289)
ILSS composite (community functioning)	0.228 (0.034)	0.263 (0.015)	−0.154 (0.157)	−0.143 (0.190)	−0.173 (0.10)
SLOF informant composite (community functioning)	0.076 (0.502)	0.082 (0.468)	−0.114 (0.312)	−0.087 (0.441)	−0.081 (0.375)
BPRS-positive symptoms	0.144 (0.185)	0.109 (0.319)	−0.002 (0.984)	−0.033 (0.762)	−0.075 (0.493)
CAINS total (negative symptoms)	−0.351 (0.001)	−0.352 (0.001)	0.290 (0.007)	0.218 (0.044)	0.230 (0.033)
SANS total (negative symptoms)	−0.253 (0.019)	−0.257 (0.017)	0.231 (0.032)	0.224 (0.038)	0.207 (0.056)
Calgary depression rating scale total	0.053 (0.629)	0.057 (0.603)	−0.016 (0.883)	0.026 (0.813)	0.045 (0.685)

Spearman Rho coefficient (*p* value)

*MCCB* MATRICS consensus cognitive battery, *SLOF* specific level of functioning, *CAINS* calgary assessment interview for negative symptoms, *ILSS* independent living skills survey; *SANS* scale for the assessment of negative symptoms, *BPRS* brief psychiatry rating scale. These exploratory analyses adjusted *p* values based on Bonferroni correlation (0.05/7 tests per metric = 0.007)

## Discussion

We investigated GPS-derived metrics of mobility in people with schizophrenia and found, as hypothesized, that people with schizophrenia spent more time at-home, traveled shorter distances overall and shorter distances away from home over 1 week of sampling than healthy comparators, and the magnitude of these differences were large. GPS indicators of mobility were also strongly associated with week- and day-aggregated reports of time spent at-home as measured by concurrent EMA derived responses, supporting convergent validity. Within the sample of people with schizophrenia, we found that less distance traveled from home and overall distance traveled (but not the percent of EMA surveys sampled at-home) was significantly but modestly associated with greater negative symptoms, in particular diminished motivation symptoms. There was a weaker correlation between community functioning on the ILSS, but not the SLOF. These relationships appeared to be nonlinear, with the strongest association at the lowest distance traveled. Other illness-related factors, including cognitive ability, positive and depressive symptoms or neurocognitive ability were unrelated to GPS metrics. Our findings indicate that objective passive sensing of mobility could differentiate people with schizophrenia and healthy comparators, and that among persons with schizophrenia negative symptoms are related to diminished mobility.

The symptom correlates of GPS-derived mobility were notably specific to negative symptoms, whereas positive symptoms, neurocognitive performance and depression were not associated. Thus, GPS mobility seemed most associated with what people do and their motivation to do it, vs. their capacity to perform functional tasks. This may account for the significant association between GPS mobility and the ILSS, which focus on current functional participation (e.g., current employment which was correlated with mobility) vs. the SLOF, which asks informants to appraise the individual’s abilities within functional domains. Moreover, the selective association with diminished motivation was notable because this aspect of negative symptoms is challenging to measure, and yet is a major focus of current research is due to its strong connection to functional outcome^20^.

Motivational deficits are present in ultra-high risk and first episode patients, and persistent negative symptoms appear to be a relatively strong predictor of conversion to psychosis^21,22^. Given that an estimated 94% of persons between the ages of 18–29 own smartphones^23^, digital technology such as GPS monitoring could 1 day be a practical and scalable tool for preventive care and long-term monitoring of youth at high risk for psychosis, with indicators like the ones described in this study used to identify patients who may warrant more careful or intensive intervention. In turn, this may improve the ability to direct treatment to younger adults most likely to experience later problems, a critical need in high-risk sample monitoring given that less than half of high risk patients will go on to convert to psychotic disorders^24^. Although age was not correlated with mobility, the mean age of the sample was 51 years. Research would be needed to disentangle the changes in mobility patterns across the life course that naturally occur with aging from changes in symptoms and functional impairments that may occur from early to more chronic stages of schizophrenia. We also note that mobility research has also taken into account variation in the built environment (e.g., home locations and neighborhoods), which also may vary by age and course of illness. GPS may be a useful tool for understanding illnesses, sociodemographic factors, and environmental influences on movement patterns in schizophrenia. In addition, the characteristics and acceptability of GPS data would need to be evaluated in at-risk samples, and within-persons changes in such indicators would need to be understood. Even more importantly, stakeholder engagement would be required to align data collection protocols to consumer preferences about privacy, informed consent, and user control associated with such monitoring.

These results only evaluated three person-averaged metrics derived from GPS, and many others are possible. Our study found that distance from home and distance traveled overall were slightly more sensitive to negative symptoms and functioning in schizophrenia than was the GPS-derived rate of home location (as well as more sensitive than EMA reports of location), and that these indicators may be less sensitive to impairment at greater levels of distance in that nonlinear associations were observed, as displayed in the Figures. It may be that between person variability in distances between home location and external activities may diminish the ability to detect symptom effects beyond more severely restricted individuals. We found that greater number of accurate samples was associated with reduced mobility. This, while in need of replication, may indicate that the discriminating ability of GPS may lessen at higher levels of mobility (although we note the overall healthy comparator and schizophrenia group comparisons were large). It may be that integration of GPS data with other mobile sensors on the device, such as accelerometer data, could enrich the measurement of the mobility construct across the range of levels of mobility. Additional indicators derived from GPS could be more sensitive across the range of activity, including two-dimensional indicators such as the area traveled around the home, as well as trips to and from home within the day^25^. By integrating graphical information systems, variability in the kinds of locations that people travel to and from, modes of travel, as well as the influence of the individual’s neighborhood and surroundings on mobility could be examined^26^. Temporal aspects of data can also be investigated, with instability or stability in mobility and “bouts” of home time being possible to assess. Thus, the present findings may constitute a starting point for a deeper understanding of GPS data on mobility in schizophrenia.

Among the limitations of this study, the sample was comprised of a convenience sample of comparators and outpatients willing to consent to GPS data collection and participation in EMA research. Although the sample composition was similar to that of other research studies, the results may not generalize to more severely ill participants or comparisons with healthy comparators who were working full time on average. Moreover, the duration of GPS sampling was restricted to one week and a key challenge for GPS is in reducing noise to obtain stable estimates. Noise in the data can derive from variation in the accuracy of the coordinate locations, natural day-to-day variability in distances traveled or home locations, and variability in the frequency of data collection. The standard deviations between- and within persons were large for GPS indicators, and it may be that a longer period of observation could have reduced this variability and generated more stable estimates. In data collection and processing, we found little standardized guidance on the optimal sampling rate, level of accuracy, and aggregation of data. Without such guidance, we would anticipate study-to-study variability in methods and, ultimately, diminished replicability. In this study, GPS was externally validated by patient reports of location on EMA. This also allowed us to restrict data processing to periods in which participants were actively responding to surveys and therefore likely to be carrying the device with them. Without such external validation, passive sensing could provide misleading data on location. Nonadherent behavioral patterns could be intuited from the data stream, such as prolonged periods of lack of movement, yet this would require significant amount of post-processing in the absence of concurrent EMA data.

In conclusion, our study provides initial validation of GPS-derived indicators of distance traveled as a potentially useful tool for measurement of mobility in schizophrenia and suggests that this tool could complement assessment of negative symptom severity and motivational deficits. With the advantages of passive sensing in low participant burden and ability to assess real-world, real-time patterns over extended periods, future research in GPS and other passive sensors could open new research avenues in target specification for early identification and monitoring in high-risk groups, as well for evaluating longer-term impact of preventative or functional remediation interventions. To establish GPS as a reliable and valid tool, however, standardization of data collection and processing protocols, integration of participant preferences surrounding privacy, and study designs that enable understanding of psychometric characteristics such as sensitivity to change are crucial.

## Methods

### Participants

The study protocol is described in detail elsewhere^27^. Briefly, inclusion criteria for participants were: (1) structured clinical interview for DSM-5^28^ confirmed schizophrenia or schizoaffective disorder or Healthy Comparators with no history of past or current mood, anxiety, or psychotic disorders; (2) age 18–65 (3) fluent in English; (4) able to give valid informed consent; (5) history of head trauma with loss of consciousness longer than 15 min; (6) history of seizure disorder; (7) history of stroke or dementia; (8) substance dependence in the past year; (9) sensory limitations including vision uncorrectable to 20/40 or color blindness or hearing loss that interferes with assessment. In addition, for the participants with schizophrenia, inclusion criteria were (1) able to identify one informant who agreed to provide real-world functioning ratings; (2) taking antipsychotic medication(s) in the schizophrenia group, and (3) no medication changes in the prior month. People with schizophrenia were recruited from flyers posted in board-and-cares/assisted living residences, mental health clinics and clubhouses in the UC San Diego Health, San Diego County Mental Health and Veterans Affairs San Diego Healthcare Systems. Healthy Comparators (*N* = 61) were recruited using advertisements in free periodicals and flyers posted in hospital clinic (e.g., primary care) settings. We attempted to match age and gender of healthy comparators to that of the sample with schizophrenia. Data collection occurred between 2014 and 2016. The study was approved by VA San Diego Institutional Review Board, and participants were informed about the collection of GPS data concurrent to GPS sampling and provided written informed consent.

### Procedures

All study participants completed laboratory assessments (detailed below) and, on the same day, were provided with a Samsung smartphone with Android OS, which was used to deliver EMA surveys and collect GPS coordinates. Survey assessments started on the day following the in-person visit. GPS coordinates were collected every five minutes, 24 h a day, over 7 days using an Android application GPS Logger app. In addition, the device was programmed using Samplex software to administer EMA surveys seven times per day for 7 days^27^. The EMA signals occurred at stratified random intervals that vary from day-to-day within, on average, 1.5-h windows starting at approximately 9:00 a.m. and ending at 9:00 p.m. each day. All data entries were time-stamped, and the program only recorded responses provided within a 15-min period following the signal. To encourage EMA adherence and returning the device, all participants were paid an additional $1 per EMA survey ($49 maximum; with a running total displayed on the device after each survey). All payments for in-lab assessments and EMA adherence were made when participants returned the device to the research center after the week of sampling.

EMA Surveys (see ref. ^27^ for the full protocol) were predominantly check-box questions asking about the amount of time spent at-home vs. away and functioning behaviors performed during the *past hour*, including work/school, self-care, home-care, at-home and outside-home leisure, transportation, and treatment engagement activities, as well as number of social interactions and the concurrent social context (e.g., family, friends, roommates, strangers, and co-workers/classmates). The specific EMA question of focus for this study was, “In the past hour, how much time did you spend at home?” Response options were on a visual analog scale from 0, 15, 30, 45, or 60 min, and we evaluated the mean response to this question and the number of times 60 min of at home time was endorsed.

### In lab measures

#### Functional outcome

Functioning was assessed using the ILSS^29^ and the SLOF^30^. The ILSS is a 51-item, self-report measure that assesses functioning behaviors across ten domains over the last month. The SLOF scale is an informant-rated report of a patient’s behavior and functioning in the following domains: Interpersonal relationships, Participation in Community and Household Activities, and Work Skills. The SLOF’s Physical Functioning, Self-Care and Socially Acceptable Behavior subscales were not used because they did not assess everyday functioning.

#### Neurocognition

The MATRICS consensus cognitive battery (MCCB^31^) was used to assess neurocognition. A global neurocognitive T-score was computed as the mean of all demographically-adjusted T-scores from the six domains of cognition, including: speed of processing, attention/vigilance, verbal memory, visual memory, working memory, and reasoning and problem solving (excluding social cognition).

#### Symptoms

The SANS^32^ and CAINS^33^ were used to measure negative symptoms. The positive symptom subscale of the expanded brief psychiatric rating scale (BPRS^34^) was used to assess positive symptoms. In addition, the Calgary Depression Scale^35^ was used to measure depressive symptomatology.

### GPS data processing

The phone produced seven XML-based GPS Exchange Format files, one for each of the seven 24-h sampling periods. Longitude, latitude, and tracking variables were recorded every 5-min, producing a maximum of 288 samples per day. XML files were converted to plain-text CSV files to allow for concatenation of samples, per subject, using R Studio scripts. In addition to coordinates, GPS receivers produce an estimated positional error, an indicator of the estimated precision of the coordinates that is expressed in distance. Accuracy of GPS signals depends upon many factors, including the positioning of the receiver relative to satellites, interference, such as structures, weather/atmospheric conditions, and the GPS sensor itself. We were unable to identify literature that provided a standardized criterion for minimum accuracy for our purposes. We, therefore, selected 100 ft as a reasonable approximation of the size of a typical home setting. The concatenated data was cleaned by removing samples with accuracy (precision of estimated location) values greater than 100 ft. Speed and bearing variables were not examined. Timestamps were used to create a separate raw-hour variable that allowed the dataset to be filtered into two categories: daytime (6 a.m. to 1 a.m.) and nighttime (2 a.m. to 5 a.m.). The mode latitude and longitude from the “nighttime” values were calculated for each participant and logged in a master data set. Mode values during nighttime hours were presumed to be the most viable method of detecting the likely “home” location for the participant. These mode values were then compared to each sample of the daytime coordinates to derive a difference score (in feet), which were later averaged by day and across the entire week. Due to nonnormal distribution of daily values, medians values were used. The process of calculating the distance between coordinate points required the use of Excel-based macros, which applied the Haversine WGS84 formula (*a* = sin^2^(Δ*φ*/2) + cos*φ*_1_ × cos*φ*_2_ × sin^2^(Δ*λ*/2)) to each serially sampled GPS coordinate, producing an absolute distance change from one point to the next.

Despite efforts to encourage regular charging of the devices, review of the raw data demonstrated that in some cases the device recorded less than the total possible of 288 samples per day. Any participant data with less than seven days of data were excluded from the study and any day with less than 20 samples was also removed. The number of samples per day, per participant, was calculated and used to adjust the average distance calculations in order to limit the error introduced by sample frequency variances. After applying these restrictions to the GPS data, a total of five healthy comparator and five patients were not included in the analyses. Visual inspection of the distribution of results revealed that there were two outlier cases in the group with schizophrenia, with median distance traveled of 72.2 and 75.5 miles which exceeded the common criterion of four standard deviations^36^ higher than the subgroup mean of 9.5 miles (sd = 13.3). We therefore removed these outliers, and so the sample analyzed included 86 people with schizophrenia and 56 healthy comparators.

### Statistical analyses

We first checked the distribution of variables and GPS values to see if they were nonnormally distributed (skewness or kurtosis >±2) and applied nonparametric statistics where necessary. We also examined the association of GPS sample counts on distance and time at-home variables, and we contrasted these across diagnostic groups; we later included GPS sample count as a covariate. Concurrent validity of EMA and GPS was assessed in two ways: (1) evaluation of the Spearman correlations between person-aggregated EMA time spent at-home and GPS indicators, and (2) evaluation of the day-to-day association between daily GPS and EMA values using mixed-linear models, with subject as a random effect. We included in these mixed models a main effect for diagnosis and an interaction-term for diagnosis and GPS value to determine if the association between EMA and GPS varied across diagnoses. Comparisons between healthy comparators and schizophrenia were performed by way of Kolmogorov Z-tests, and Cohen’s d effect sizes were calculated for these comparisons. We repeated these analyses with generalized linear models with significant group demographic differences as covariates, as well as differences in sampling rate. Finally, we evaluated the association between demographic and in-lab measures and GPS metrics using Spearman correlations. The *p* value for the study was set to 0.05, and for exploratory analyses we adjusted *p* values based on Bonferroni correlation (0.05/7 tests per metric = 0.007).

### Reporting summary

Further information on research design is available in the Nature Research Reporting Summary linked to this article.

## Supplementary information

Reporting Summary Checklist

## Data Availability

These data are available to investigators upon request and pending eligibility to access data governed by the VA San Diego Institutional Review Board.
